# COMT genetic variation confers risk for psychotic and affective disorders: a case control study

**DOI:** 10.1186/1744-9081-1-19

**Published:** 2005-10-18

**Authors:** Birgit Funke, Anil K Malhotra, Christine T Finn, Alex M Plocik, Stephen L Lake, Todd Lencz, Pamela DeRosse, John M Kane, Raju Kucherlapati

**Affiliations:** 1 Harvard Partners Center for Genetics and Genomics, Boston, USA; 2 Psychiatry Research, The Zucker Hillside Hospital, Glen Oaks, NY, USA; 3 Channing Laboratory, Brigham and Women's Hospital, Boston, USA

## Abstract

**Background:**

Variation in the COMT gene has been implicated in a number of psychiatric disorders, including psychotic, affective and anxiety disorders. The majority of these studies have focused on the functional Val108/158Met polymorphism and yielded conflicting results, with limited studies examining the relationship between other polymorphisms, or haplotypes, and psychiatric illness. We hypothesized that COMT variation may confer a general risk for psychiatric disorders and have genotyped four COMT variants (Val158Met, rs737865, rs165599, and a SNP in the P2 promoter [-278A/G; rs2097603]) in 394 Caucasian cases and 467 controls. Cases included patients with schizophrenia (n = 196), schizoaffective disorder (n = 62), bipolar disorder (n = 82), major depression (n = 30), and patients diagnosed with either psychotic disorder NOS or depressive disorder NOS (n = 24).

**Results:**

SNP rs2097603, the Val/Met variant and SNP rs165599 were significantly associated (p = 0.004; p = 0.05; p = 0.035) with a broad "all affected" diagnosis. Haplotype analysis revealed a potentially protective G-A-A-A haplotype haplotype (-278A/G; rs737865; Val108/158Met; rs165599), which was significantly underrepresented in this group (p = 0.0033) and contained the opposite alleles of the risk haplotype previously described by Shifman et al. Analysis of diagnostic subgroups within the "all affecteds group" showed an association of COMT in patients with psychotic disorders as well as in cases with affective illness although the associated variants differed. The protective haplotype remained significantly underrepresented in most of these subgroups.

**Conclusion:**

Our results support the view that COMT variation provides a weak general predisposition to neuropsychiatric disease including psychotic and affective disorders.

## Background

The role of Catechol-O-Methyltransferase (COMT) in dopamine metabolism has led to investigation of its variants in the etiology of numerous psychiatric disorders including psychotic, affective and anxiety disorders. The largest body of work exists for schizophrenia and bipolar disorder because **1. **Imbalance of dopamine is thought be key to the pathogenesis of psychosis [1,2], **2. **COMT is located in the region on chromosome 22q11 commonly deleted in velo-cardio-facial/DiGeorge syndrome (VCFS/DGS) whose phenotypic spectrum includes severe psychiatric disease that has been described as schizophrenia by some [3-5] and bipolar disorder by others [6] and **3. **Genetic variation in COMT has been implicated in prefrontal cortical function [7,8], which is commonly impaired in both disorders [9]. In addition to schizophrenia and bipolar disorder, evidence for a contribution of COMT variants exists for panic disorder [10,11], attention deficit hyperactivity disorder [12], obsessive compulsive disorder [13], phobic anxiety [14] and anorexia nervosa [15].

Most studies focused on a common functional SNP (Val108/158Met) because the Methionine-containing variant shows a significant reduction in enzyme activity [16-18]. However, despite their large number, these studies have generated controversial and confusing results: For schizophrenia, initial studies have reported an association of the A (Met) allele [19-21]. However, current evidence favors an association of the of the G (Val) allele [22-27]. Similarly, for bipolar disorder, several studies reported an association of Met-COMT [[28-31], reviewed in: [32]]; but a recent study by Shifman et al. showed evidence of an association of Val-COMT [33]. As with schizophrenia and bipolar disorder, the associations remain controversial for other psychiatric illnesses including ADHD, OCD, anorexia nervosa, and anxiety disorder [34-37]. In all cases, likely contributing factors are small sample sizes and/or diagnostic differences as the absence of objective biomarkers in psychiatric disorders potentially hampers consistent classification of disease [38,39].

Although Shifman et al. saw an association of the Valine allele in their cohort, its moderate effect combined with highly significant p-values for two SNPs located in intron 1 and the 3'UTR (rs737865 and rs165599) has led to the hypothesis that the Val/Met variant may not contribute to disease but may simply be in strong LD with the actual, as of yet unidentified pre-disposing variant [40]. A recent study by Handoko et al. supports this view [41].

The COMT gene is transcribed from two promoters resulting in a cytoplasmatic form (soluble; S-COMT, transcribed from P1) and a membrane bound form (MB-COMT, transcribed from P2) [42]. Although both variants are widely expressed at varying levels, MB-COMT appears to be the predominant from in brain [42,43]. It has therefore been suggested that disease pre-disposing variant/s may be located in the P2 promoter, acting in cis to alter COMT protein levels via enhancement or suppression of transcription [44]. Several recent studies have investigated the effect of previously associated variants on COMT expression levels [16,45-47]. Interestingly, some showed reduced expression levels of Valine-coding COMT mRNAs [45,47]. Although this contrasts with the higher enzyme activity of Val-COMT, the net result of these two effects seems to be a 40% higher enzyme activity in human dorsolateral prefrontal cortex samples homozygous for Val-COMT [16]. A variant located in the P2 promoter (-278A/G) showed a small effect on enzyme activity, suggesting that it may indeed influence brain dopamine levels [16].

Taken together, current evidence suggests that COMT variants may provide a weak predisposition to a variety of psychiatric conditions via alteration of dopamine levels in the prefrontal cortex (supported by the association of the same haplotype with both, schizophrenia and bipolar disorder) [33]. Expression of specific disorders may require the presence of additional predisposing variants in susceptibility genes specific for these pathologies. Therefore, we have tested the relationship between COMT variation and psychiatric illness in a large cohort of Caucasian patients. We included subjects with a range of psychiatric diseases, and genotyped 4 SNPs including the Val/Met polymorphism, the P2 promoter SNP (-278A/G, rs2097603) as well as SNPs rs737865 and rs165599 to test the hypothesis that COMT genetic variation is associated with the risk for psychiatric illness. We analyzed the relationship between four-marker haplotypes, as well as the individual SNPs. Moreover, we conducted exploratory analyses of specific diagnostic subgroups within the cohort to assess the relationship between COMT and various psychiatric diagnoses.

## Methods

### Study Subjects

The sample was comprised of 394 US-Caucasian cases (mean age = 39.4 years; 35.7% females/64.3% males). Diagnostic categories included schizophrenia (n = 196), schizoaffective disorder (n = 62), bipolar disorder (n = 82), major depression (n = 30), psychotic disorder NOS (19) and depressive disorder NOS (5). The control group consisted of 467 Caucasian individuals (mean age = 39.3 years, 51.8% females/48.2% males). **Cases **were recruited from the inpatient and outpatient clinical services of the Zucker Hillside Hospital, a division of the North Shore – Long Island Jewish Health System, where patients are screened for potential recruitment into research studies by the Clinical Assessment and Training Unit (CAT) of the NIH-funded Hillside Hospital Intervention Research Center. The CAT monitors the inpatient and outpatient hospital census daily and conducts preliminary screening and recruitment functions. Inclusion criteria for screening for this study included a clinical diagnosis of a psychotic disorder, no active substance abuse, and ability to provide informed consent. After obtaining written informed consent, each subject was assessed with the Structured Clinical Interview for DSM-IV Axis I Disorders (SCID: version 2.0, 8/98), administered by trained raters. Standardized diagnostic assessments were supplemented with clinical information obtained by review of medical records and interviews with family informants when possible, and all diagnostic information was compiled into a narrative case summary. Information on the onset and course of Axis I illness, presence of Axis II pathology, presence of Axis III diagnoses, and a brief description of the subject's psychosocial and occupational functioning during the course of illness was presented to a consensus diagnostic committee consisting of a minimum of three senior faculty with DSM-IV diagnostic experience, as well as other faculty and trainees with SCID experience. All available information was used to arrive at a consensus DSM-IV diagnosis. **Healthy controls **were ascertained and recruited by the Zucker Hillside Hospital Normal Control Program. Potential participants were recruited via local newspaper advertisements, flyers and community internet resources and underwent initial telephone screening to assess eligibility criteria. Subjects meeting eligibility criteria were administered the SCID – NP to rule out the presence of an Axis I psychiatric disorder, urine toxicology screen for drugs of and a family history of psychiatric disorder assessment. Exclusion criteria included current or past: Axis I psychiatric disorder, psychotropic drug treatment, substance abuse, first-degree family member with an Axis I psychiatric disorder, or inability to provide written informed consent. A subset of the control cohort was collected through the Massachusetts General Hospital Clinical Research Program in conjunction with the Harvard-Partners Center for Genetics and Genomics in Boston, MA. This subset comprised disease free subjects over the age of 18. For the purposes of the study, "disease" was defined as current or past diagnoses made by a medical care provider that required medication or other forms of treatment/therapy. With the exception of orthopedic procedures, appendectomy, or those that were trauma related, surgery was considered an exclusionary criterion. Subjects who took prescription medications or regularly used over the counter medications were also excluded. These subjects completed a structured family and medical questionnaire that detailed current and past history of psychiatric illness and pharmacological or psychotherapeutic psychiatric treatment. All responses to the self-report form were confirmed by clinical interview by physicians. Physical exams were completed at the study visit. This study was approved by the Institutional Review Board at NSLIJHS and Partners Healthcare.

### Genotyping

All samples were genotyped using the ABI PRISM 7900HT Sequence Detection System (Applied Biosystems, Foster City, CA). The 5' nuclease assay (TaqMan^®^) was used to distinguish the two alleles of a gene. PCR amplification was carried out on 5–20 ng DNA using 1 × TaqMan^® ^universal PCR master mix (No Amp-erase UNG), 900 nM forward and reverse primers, 200 nM of the FAM labeled probe and 200 nM of the VIC labeled probe in a 5 ul reaction volume. Amplification conditions on an AB 9700 dual plate thermal cycle (Applied Biosystems, Foster City, CA) were as follows: 1 cycle of 95°C for 10 min, followed by 50 cycles of 92°C for 15 s and 58°C for 1 min. TaqMan^® ^primers and probes were designed using the Primer Express^® ^Oligo Design software v2.0 (ABI PRISM) or using the ABI Assays-By-Design service.

### Statistical Analyses

Tests of Hardy-Weinberg equilibrium (HWE) were performed at each SNP locus [48]. Application of the test for HWE was restricted to the control samples from each ethnic group as a means of identification of genotyping problems. *Single SNP analyses: *Statistical inference for single SNP associations was based on Chi-square test statistics. For each SNP an allelic association test and the Cochran-Armitage trend test (test for additive allelic effects) were performed [49]. *Haplotype analyses: *Haplotype associations were explored using score tests that account for linkage phase ambiguity [50]. The score tests, derived from generalized linear models, are used for global tests of association, as well as haplotype-specific tests. In addition, a permutation algorithm was applied in the testing framework to find the maximum of the haplotype-specific score statistics and its associated p-value. The *haplo.stats *program implements the methods of Schaid *et al*. [50] and was used for these analyses. Haplotypes were imputed and frequencies estimated using the EM-algorithm-based estimation facility in *haplo.stats*.

## Results

### Single SNP analyses

We tested four COMT SNPs (-278 A/G, rs737865, Val108/158Met and rs165599) for association with diagnosis in our sample of 394 US-Caucasian cases and 467 controls. All SNPs were in HWE (data not shown). The results of the single SNP analysis are presented in Table 1. Three of the four SNPs were significantly associated with our broad "all affecteds" diagnosis: -278 A/G (p = 0.004; OR = 1.34), Val108/158Met (p = 0.05; OR = 1.21) and rs165599 (p = 0.035; OR = 1.25). When the cases were restricted to patients with schizophrenia or schizoaffective disorder (n = 258), only the promoter polymorphism (-278 A/G) remained significant (p = 0.015; OR = 1.33). This SNP also remained significant when only patients with schizophrenia were included (n = 196; p = 0.011, OR = 1.39). None of the SNPs yielded significant p-values in the group of patients with schizoaffective disorder (n = 62; data not shown). In the set of patients who were diagnosed with an affective disorder (n = 112; 82 bipolar disorder, 30 major depressive disorder), significant p-values were obtained for the G (Val) allele of the Val/Met polymorphism (p = 0.018; OR = 1.43) and the G allele of rs165599 (p = 0.039; OR = 1.38). The A allele of SNP -278A/G showed a trend for significance in this group (p = 0.062; OR = 1.34). When broken down into subcategories, -278A/G was marginally associated in the major depressive group (n = 30; p = 0.046; OR = 1.79) and Val158Met showed a trend towards association in the group of bipolar patients (n = 82; p = 0.058, OR = 1.39). Overall evidence for association was most robust for the promoter polymorphism -278 A/G.

**Table 1:** Allele frequencies

		All affecteds*					**SCZ + SA**				
**SNP ID (dbSNP No)**	Alleles (major/minor) *	**MAF Controls**	**MAF Cases**	**p-value**	**Trend p-value**	**OR (95%CI)**	**MAF Controls**	**MAF Cases**	**p-value**	**Trend p-value**	**OR (95%CI)**

		**n = 467**	**n = 394**				**n = 467**	**n = 258**			

rs2097063 (287A/G)	**A**/G	0.414	0.346	***0.004***	***0.006***	1.34 (1.10, 1.64)	0.414	0.347	***0.015***	***0.019***	1.33 (1.06, 1.67)
rs737865	T/C	0.309	0.332	0.315	0.321	1.11 (0.90, 1.37)	0.309	0.329	0.438	0.448	1.10 (0.87, 1.39)
rs4680 (Val/Met)	A/**G**	0.475	0.524	***0.050***	***0.048***	1.21 (1.00, 1.47)	0.475	0.504	0.304	0.299	1.12 (0.90, 1.40)
rs165599	A/**G**	0.326	0.376	***0.035***	***0.040***	1.25 (1.02, 1.53)	0.326	0.364	0.160	0.175	1.18 (0.94, 1.49)

		**SCZ**					Affective disorder*				

**SNP ID (dbSNP No)**	Alleles (major/minor) *	**MAF Controls**	**MAF Cases**	**p-value**	**Trend p-value**	**OR (95%CI)**	**MAF Controls**	**MAF Cases**	**p-value**	**Trend p-value**	**OR (95%CI)**

		**n = 467**	**n = 196**				**n = 467**	**n = 112**			

rs2097063 (287A/G)	**A**/G	0.414	0.338	***0.011***	***0.014***	1.39 (1.08, 1.79)	0.414	0.346	*0.062*	*0.067*	1.34 (0.98, 1.82)
rs737865	T/C	0.309	0.327	0.511	0.518	1.09 (0.84, 1.41)	0.309	0.329	0.560	0.556	1.10 (0.80, 1.50)
rs4680 (Val/Met)	A/**G**	0.475	0.500	0.419	0.414	1.10 (0.87, 1.41)	0.475	0.565	***0.018***	***0.015***	1.43 (1.06, 1.93)
rs165599	A/**G**	0.326	0.362	0.221	0.240	1.17 (0.91, 1.51)	0.326	0.400	***0.039***	***0.043***	1.38 (1.02, 1.87)

		**Major depressive disorder**					**BP**				

**SNP ID (dbSNP No)**	Alleles (major/minor) *	**MAF Controls**	**MAF Cases**	**p-value**	**Trend p-value**	**OR (95%CI)**	**MAF Controls**	**MAF Cases**	**p-value**	**Trend p-value**	**OR (95%CI)**

		**n = 467**	**n = 30**				**n = 467**	**n = 82**			

rs2097063 (287A/G)	**A**/G	0.414	0.283	***0.046***	***0.049***	1.79 (1.01, 3.18)	0.414	0.369	0.280	0.290	1.21 (0.86, 1.71)
rs737865	T/C	0.309	0.333	0.688	0.689	1.12 (0.64, 1.95)	0.309	0.327	0.638	0.634	1.09 (0.76, 1.56)
rs4680 (Val/Met)	A/**G**	0.475	0.586	0.101	0.093	1.56 (0.91, 2.68)	0.475	0.557	***0.058***	***0.050***	1.39 (0.99, 1.95)
rs165599	A/**G**	0.326	0.414	0.169	0.184	1.46 (0.85, 2.51)	0.326	0.395	0.088	0.095	1.35 (0.96, 1.91)

* All Affecteds: SCZ (schizophrenia), SA (schizoaffective disorder), BP (bipolar disorder), Major depressive disorder, psychotic disorder NOS, depressive disorder NOS

* Affective Disorder: Bipolar disorder and major depressive disorder

### Marker-to-Marker Linkage Disequilibrium

Linkage disequilibrium (LD) between the four SNPs was assessed in cases and controls via Lewontin's D' statistic (Table 2). The markers span 28.5 kb on genomic DNA. In general, LD was higher between markers located in the 5' region of the gene. SNP -287 A/G is located only 2 kb from SNP rs737865 and is in complete LD with it. LD was also high for marker pair rs737865 – Val/Met. SNP rs165599 showed modest LD with the Val/Met polymorphism. This pattern is consistent with previous reports [40,45]. In contrast, a recent study by Chen *et al*. found only modest LD between rs737865 and the Val/Met polymorphism [22]. With the exception of the Val/Met – 737865 marker pair, D' values were similar in the schizophrenia/schizoaffective disorder group and the affective disorder group. In the latter, the Val/Met was in complete LD with rs737865, which may have contributed to the differences in the statistical difference observed for this SNP.

**Table 2:** Linkage Disequilibrium (D')

**Controls**	rs737865	Val/Met	rs165599
-287A/G	1	0.57	0.37
rs737865		0.72	0.23
Val/Met			0.67

**Affected**	rs737865	Val/Met	rs165599

-287A/G	1	0.51	0.27
rs737865		0.85	0.21
Val/Met			0.65

**SCZ/SA**	rs737865	Val/Met	rs165599

-287A/G	1	0.53	0.32
rs737865		0.76	0.28
Val/Met			0.64

**Affective Disorder**	rs737865	Val/Met	rs165599

-287A/G	1	0.53	0.29
rs737865		1	0.23
Val/Met			0.52

### Haplotype analyses

Shifman *et al*. described a highly significant association of a three-site haplotype consisting of the G allele of SNPs rs737865, Val/Met and rs165599. This haplotype was associated with schizophrenia and bipolar disorder [33,40]. We constructed four-marker haplotypes for the Caucasian group including -278 A/G and the three SNPs present on the Shifman haplotype. Ten haplotypes had estimated frequencies above 0.025 and were included in the association testing (Table 3). This large number is most likely due to the low LD of rs165599 with the other three markers. One haplotype (G-A-A-A) was significantly underrepresented in cases and could be protective (p = 0.0033; maximum haplotype specific p = 0.026). Haplotype frequencies of the "opposite" haplotype (A-G-G-G), which encompasses the "Shifman haplotype did not differ significantly in the all affecteds group (Table 3), or any of the subgroups (data not shown). In contrast, the potentially protective G-A-A-A haplotype remained significantly underrepresented in most subgroups (Table 4).

**Table 3:** Haplotypes observed in the "all affecteds" group

**287A/G (A/G)**	**rs737865 (A/G)**	**Val/Met (A/G)**	**rs165599 (A/G)**	**Frequency (cases)**	**Frequency (controls)**	**Haplotype-specific p-values**
G	A	A	A	0.2226	0.2809	***0.0033***
A	G	G	G	0.1763	0.1489	0.1212
A	A	A	A	0.1629	0.1446	0.2784
A	G	G	A	0.1261	0.1191	0.7400
A	A	G	G	0.0829	0.0807	0.3639
G	A	G	G	0.0548	0.0385	0.3524
A	A	G	A	0.0538	0.0386	0.2169
G	A	A	G	0.0424	0.0462	0.8213
G	A	G	A	0.0282	0.0490	0.0884
A	G	A	A	0.0300	0.0418	0.2253

**Table 4:** Frequency of the G-A-A-A haplotype

	**cases**	**controls**	**Haplotype-specific p-values**
All	0.2226	0.2809	***0.0033***
SCZ+SA	0.2282	0.2809	***0.0191***
SCZ	0.2297	0.2809	***0.0361***
SA	0.2255	0.2809	0.1641
BP + MD	0.2188	0.2809	***0.0343***
BP	0.2383	0.2809	0.1886
MD	0.1554	0.2809	***0.0378***

## Discussion

The COMT gene has been extensively studied as a candidate gene for a variety of psychiatric disorders including schizophrenia, bipolar disorder, and other psychiatric conditions because of 1. Its known function in dopamine metabolism 2. The presence of a common functional nonsynonymous SNP in exon 4 (Val/Met), which alters enzyme activity and 3. Its location in the region commonly deleted in VCFS/DGS, which is associated with severe psychiatric disease often diagnosed as schizophrenia. However, no consistent picture has yet emerged, which might in part be due to small sample size of many studies.

We hypothesized that variation at the COMT locus confers a general basic risk for developing neuropsychiatric disease and therefore genotyped several variants in a heterogeneous group of patients. Several studies support this hypothesis: Results from family, twin, linkage and association studies show an overlapping genetic etiology of schizophrenia and bipolar disorder [reviewed in: [51]]. Several "overlap genes" including G72/G30 [52-55], Neuregulin [56,57] and DISC1 [58] have now been associated with both schizophrenia and bipolar disorder.

In addition, family studies have also shown evidence for an overlap between the genetic etiologies of schizophrenia and major depressive disorder: For example, the incidence of major affective disorder (bipolar and unipolar) was increased in relatives of probands with schizophrenia or schizoaffective disorder [59]. Data from another study supports the view that there could be a familial relationship between the predispositions to schizophrenia and to major depression as SCZ probands had an increased familial risk for unipolar major depressive disorder [60].

The SNPs genotyped in our sample included the well-known Val/Met variant, two SNPs that were highly associated in a previous study (rs737865 and rs165599 [40]) as well as -287A/G, a polymorphism in the P2 promoter. Three SNPs (-278**A**/G, **Val**/Met and rs165599 A/**G**) were associated in this broad diagnostic group with -278 A/G showing the most significant p-values. Haplotype revealed the existence of a potentially protective haplotype (G-A-A-A), which was significantly underrepresented in our all affecteds category and remained significant in most analyzed subgroups. The haplotype consisting of the "opposite" alleles of the risk haplotype described by Shifman et al. [33,40] was not significantly overrepresented in our cases.

**Figure 1:**
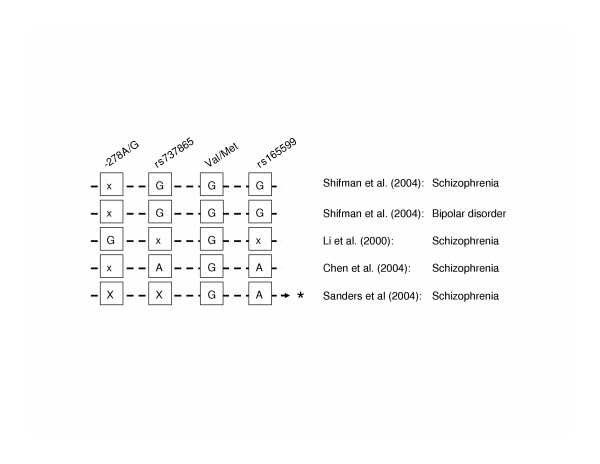
Risk haplotypes identified in different studies and populations are shown. SNP IDs are indicated on top and the respective alleles are boxed underneath. "x" denotes a SNP that was not tested. * indicates a haplotype, which also included rs9265(A) and rs165849(A), both located distal to rs165599 in the ARVCF gene.

Our results add to the growing number of studies showing an association of the Valine allele of the Val108/158 Met polymorphism, the only proven functional COMT variant so far. Interestingly, a comparison of risk haplotypes identified in previous studies showed that, even though the individual haplotypes vary with respect to the combination of alleles, all contain the Valine allele, indicating that it may be much older than their division [Figure 1 and references [22,24,27,33,40]].

In addition to the heterogeneous "all affecteds" category, we separately analyzed cases with psychotic and affective illness to analyze the contribution of the four variants to disease in these subgroups. In the psychotic group (schizophrenia and schizoaffective disorder), only the promoter variant (-287**A**/G) remained significant. The other two SNPs associated in the all affecteds group were not significant although the allele frequencies still showed an overrepresentation of the same alleles. In the affective disorder group, the association was driven by the 3' portion of the gene (Val-COMT and rs165599 A/**G**). The promoter polymorphism showed a trend for association in this group. We suggest that one or a combination of the following factors might have contributed to these results: First, it is conceivable that COMT harbors more than one functional variant. Although these may collectively confer a general risk for neuropsychiatric disease, the magnitude of the effect may vary depending on diagnosis. Support for this view comes from a recent family-based study reporting two separate and interacting effects within a haplotype spanning rs737865-Val/Met-rs165599 [41]. Second, the lack of association of some variants in the patients with affective disorder and not in the SCZ/SA group may be related to the limitations inherent in a diagnostic system based upon clinical phenomena, which may not correctly reflect the underlying biology that predisposes to illness. Therefore, in the absence of biological markers, a strict separation of our cohort into schizophrenic and affective disorder patients may be too stringent [38,39]. Third, we noted that while the affective disorder group was well matched with regard to gender (49% male, 51% female), the schizophrenia/schizoaffective group showed an excess of males (67%). This imbalance may have contributed to the lack of association of Val/Met and rs165599 as schizophrenia may have gender-specific differences [61,62] and previous studies suggested a stronger association of the G (Val) allele or a Val-containing haplotype in females [25,40]. It has also been suggested that the Val/Met variant does not contribute to disease but is merely in high LD with the actual functional variant [40,41]. We observed different D' values for the Val/Met – rs737865 marker pair in the analyzed patient subgroups which may have contributed to the differences in the statistical significance of the Val/Met SNP.

Finally, one could argue that our results were influenced by hidden population substructure, leading to false positive results. However, while this is a possible limitation of our study, empirical data obtained from US-Caucasian, African American and European populations suggest that carefully matched studies of moderate size are unlikely to contain significant stratification levels [63,64]. In particular, a recent study by Tang *et al*. showed that self-identified ethnicity is the major determinant of genetic structure in the United States [65].

## Conclusion

Our study supports a general role of the COMT gene in the genetic etiology of neuropsychiatric disease including psychotic and affective disorders. Further studies are needed to unambiguously determine the nature and specific contribution of the functional variant/s.

## List of Abbreviations used

SCZ (schizophrenia); BP (Bipolar disorder), SA (schizoaffective disorder), ADHD (Attention deficit hyperactivity disorder), OCD (obsessive compulsive disorder).

## Competing interests

The author(s) declare that they have no competing interests.
